# Preparation and Characterization of Zein/Salecan Nanocomposite Particles for Enhanced Stability and Bioactivity of Lipophilic Retinoids

**DOI:** 10.1002/asia.70741

**Published:** 2026-04-14

**Authors:** Jie Mei, Chen Li, Yunxing Li, Beizhe Chang, Hang Jiang, Cheng Yang, Bingtian Zhao, To Ngai

**Affiliations:** ^1^ Key Laboratory of Synthetic and Biological Colloids, School of Chemical and Material Engineering, Ministry of Education Jiangnan University Wuxi China; ^2^ School of Chemistry and Environmental Engineering Yuxi Normal University Yuxi China; ^3^ Department of Chemistry The Chinese University of Hong Kong Shatin Hong Kong China

**Keywords:** delivery, encapsulation, nanocomposite particle, salecan, zein

## Abstract

Retinoids are lipophilic compounds with high biological activity but poor stability and water solubility. Herein, novel zein/salecan (Zein/Sal) nanocomposite particles were fabricated via antisolvent precipitation with polysaccharide coating and used as carriers for hydroxypinacolone retinoate (HPR). The mass ratio of zein to Sal significantly affected particle properties, with a mass ratio of 2:1 yielding a homogeneous spherical morphology and high encapsulation efficiency, while HPR was integrated in an amorphous state. The integration of zein with HPR was due to hydrogen bonding and hydrophobic interactions. The resulting HPR‐loaded Zein/Sal nanocomposite particles exhibited good colloidal stability over a wide pH range, under high ionic strength conditions, and during long‐term storage. Encapsulation also remarkably improved HPR stability under different temperatures and light irradiation. In vitro assays revealed that the Zein/Sal nanocomposite particles had good biocompatibility, reduced the cytotoxicity of encapsulated HPR, and promoted cellular uptake and migration with the encapsulated HPR. Steady release and improved transdermal delivery of encapsulated HPR were also achieved. These results show that Sal serves as a functional stabilizer, improving both the structural and biological functions of zein nanoparticles, and offering a viable vehicle for the delivery of lipophilic retinoids in food, pharmaceutical, and cosmetic applications.

## Graphical abstract

Zein/Salecan nanocomposite particles were developed to enhance the stability and functionality of zein nanoparticles, and they exhibited good encapsulation of lipophilic hydroxypinacolone retinoate (HPR), superior light, thermal, and storage stability, reduced cytotoxicity, and enhanced cell migration rate and cellular uptake of the encapsulated HPR, and the ability to release HPR steadily in vitro without compromising its skin permeability. The graphic was created with BioRender (http://biorender.com).

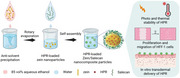

## 1 Introduction

Retinoids, a group of lipophilic compounds such as retinol, retinal, retinoic acid, and their derivatives, are characterized by a conjugated polyene chain that provides both high biological activity and chemical instability [1]. They affect essential physiological processes such as eyesight, immunological response, cell differentiation, and tissue homeostasis, and are widely used in pharmaceuticals, food, and cosmetics [2, 3, 4, 5, 6]. However, like most lipophilic bioactives, the significant hydrophobicity and sensitivity to light, oxygen, heat, and metal ions result in reduced efficacy and bioavailability [7, 8, 9]. To address these issues, encapsulation of these bioactives in nanoscale carriers has emerged as a promising strategy to increase their stability, solubility, and, in some cases, biocompatibility.

Zein is a natural prolamin extracted from corn that has been recognized as Generally Recognized as Safe by the US Food and Drug Administration [10]. Its amphiphilic nature allows it to self‐assemble into zein nanoparticles (ZNPs) via antisolvent precipitation. ZNPs have been intensively investigated as a biocompatible and biodegradable vehicle for hydrophobic bioactives [11, 12]. Lutein, curcumin, and paclitaxel, for example, have been successfully encapsulated in ZNPs, resulting in improvements in their solubility, stability, and biological activity [13, 14, 15]. Nevertheless, the application of ZNPs is limited by their low colloidal stability near neutral pH, as zein has an isoelectric point around 6.0, leading to flocculation and aggregation under physiological conditions [16]. To address this limitation, different polysaccharides have been used to stabilize ZNPs, with polysaccharides providing electrostatic and steric stabilization [17]. For example, xanthan gum, hyaluronic acid, and pectin stabilized ZNPs have demonstrated improved resistance to pH and ionic changes [16, 18, 19]. However, most research has concentrated primarily on the stabilizing role of polysaccharides, ignoring the possibility of using functional stabilizers that not only improve colloidal stability but also offer biological activities that synergize with those of the encapsulated bioactives.

*β*‐glucan, a naturally occurring polysaccharide found in bacteria, fungi, yeast, and cereals, is biocompatible and biodegradable. Various sources of *β*‐glucan have different structures and molecular weights, resulting in varying physicochemical properties and biological activities, including immune‐modulation, anti‐inflammatory, anti‐aging, and cell proliferation [20, 21]. Microbial *β*‐glucan is typically faster and simpler to produce than other natural sources [22]. However, most of these, such as curdlan and yeast *β*‐glucan, are often poorly soluble or insoluble in aqueous solutions [23, 24]. Chemical modifications, such as carboxymethylation, sulfonylation, and amination, can enhance solubility and stabilize hydrophobic nanoparticles by generating water‐soluble derivatives of *β*‐glucan [21]. For example, Bao et al. prepared zein‐yeast carboxymethyl glucan nanocomposite particles using an ultrasonically assisted anti‐solvent procedure, which exhibited improved colloidal stability and effectively encapsulated resveratrol [25]. Nevertheless, chemical modifications are complicated and may involve harmful reagents or toxic residues. Salecan (Sal), an intrinsically water‐soluble *β*‐glucan, is produced by *Agrobacterium* sp. ZX09 strain [26]. It possesses a high molecular weight and a linear backbone of D‐glucose units connected by *β*‐(1,3) and *α*‐(1,3) glycosidic bonds. Additionally, the presence of succinyl and pyruvyl substituents imparts negative charges upon ionization in aqueous media, offering electrostatic repulsion [27, 28]. These structural features are expected to endow Sal with excellent stabilizing capacity, offering both steric and electrostatic stabilization for hydrophobic substrates. To date, however, its potential as a functional stabilizer for ZNPs has not yet been explored.

In this work, Zein/Sal nanocomposite particles were developed via antisolvent precipitation of zein, followed by Sal coating. The influence of Sal concentration on the preparation of the nanocomposite particles was first investigated. The optimal Zein/Sal nanocomposite particles were then used as functional carriers for lipophilic retinoids, with hydroxypinacolone retinoate (HPR) selected as the model compound. HPR is an ester derivative of retinoic acid with high biological activity but poor water solubility and chemical stability [29]. The colloidal stability of HPR‐loaded Zein/Sal nanocomposite particles and the stability of encapsulated HPR were examined under various environmental conditions. Finally, in vitro assays were carried out to assess the cytotoxicity, cell migration‐promoting ability, cellular uptake, release behavior, and skin penetration of HPR encapsulated in Zein/Sal nanocomposite particles.

## 2 Results and Discussion

### 2.1 Formation and Characterization of Zein/Sal Nanocomposite Particles

**FIGURE 1:**
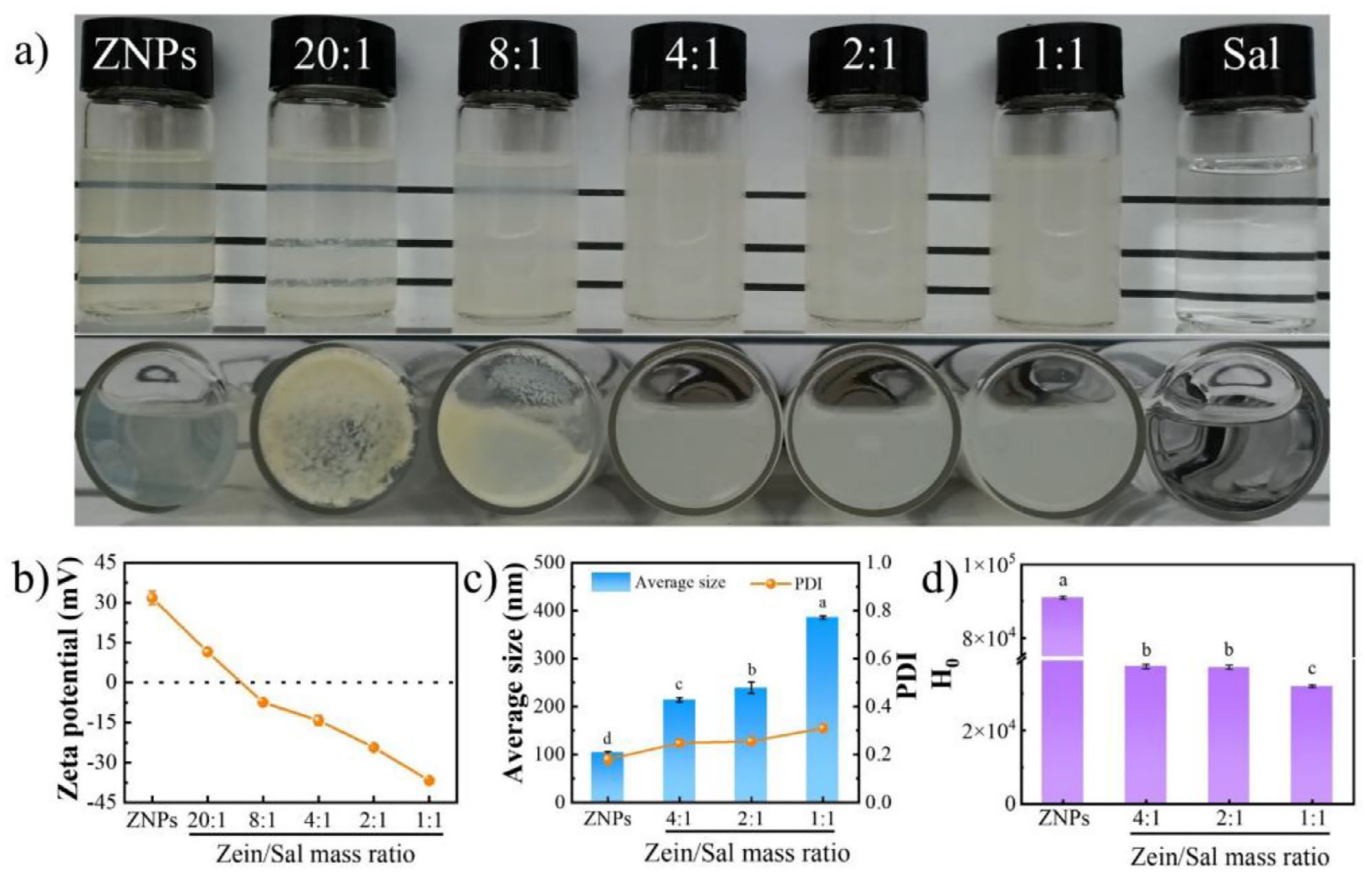
Digital images of aqueous dispersions of ZNPs and various Zein/Sal nanocomposite particles, as well as an aqueous solution of Sal, at pH 4.0 (a); zeta potential (b), average size and Polydispersity Index (PDI) (c), surface hydrophobicity index (H_0_) (d) of ZNPs and a series of Zein/Sal nanocomposite particles. Different letters represent significant differences (*p* < 0.05).

Zein readily dissolves in aqueous ethanol but precipitates upon dilution with water [30]. As a result, the antisolvent precipitation approach is commonly employed to prepare ZNPs. As the ethanol concentration decreases, zein self‐assembles into nanoparticles. Figure 1a shows that the aqueous dispersion of resulting ZNPs was homogeneous and translucent, whereas the aqueous solution of Sal was clear and colorless. When ZNPs were mixed with Sal in water, Sal was adsorbed onto their surfaces, forming Zein/Sal nanocomposite particles. The visual appearance of aqueous dispersions of Zein/Sal nanocomposite particles varied dramatically with the Sal concentration. At Zein/Sal mass ratios of 20:1 and 8:1, large aggregates were observed at the bottom of both vials, especially at the lower Sal concentration. When the mass ratio fell below 8:1, in contrast, stable and homogeneous aqueous dispersions of Zein/Sal nanocomposite particles were obtained.

Zeta potential measurements were then used to illustrate this assembly behavior (Figure 1b). ZNPs were positively charged at pH 4.0, whereas Sal carried a negative charge [31]. Therefore, electrostatic attraction between these oppositely charged components would induce the formation of Zein/Sal nanocomposite particles. At low Sal concentrations (Zein/Sal_20:1_ and Zein/Sal_8:1_), adsorption of a minor amount of Sal resulted in charge neutralization, lowering the absolute zeta potential of resulting Zein/Sal nanocomposite particles and weakening electrostatic repulsion, thus promoting particle aggregation. As the amount of Sal increased, more Sal deposited on the surface of ZNPs, leading to a higher zeta potential and thus increased electrostatic repulsion. When the Zein/Sal mass ratio reached 2:1, the zeta potential of the resulting nanocomposite particles approached ‐30 mV, indicating they would have adequate colloidal stability. Consistent with the preceding results, the average size of Zein/Sal nanocomposite particles increased as Sal concentration increased (Figure 1c), with the exception of Zein/Sal_20:1_ and Zein/Sal_8:1_. The increased adsorption of Sal on the surface of ZNPs contributed to an increase in the average size of the obtained Zein/Sal nanocomposite particles, as well as a broader particle size distribution. Furthermore, the surface hydrophobicity was evaluated using ANS, a fluorescent probe that specifically binds to the hydrophobic areas of proteins [32]. As shown in Figure 1d, ZNPs have a high surface hydrophobicity index (H_0_) of 90980.50, which corresponds to a high fraction of hydrophobic amino acids in zein [33]. In contrast, Sal is a water‐soluble polysaccharide with a H_0_ of 359.47. With the increase in Sal content, the H_0_ of the resulting nanocomposite particles gradually decreased, suggesting that the hydrophilic Sal was successfully coated on the surface of ZNPs and effectively shielded their hydrophobic surfaces.

**FIGURE 2:**
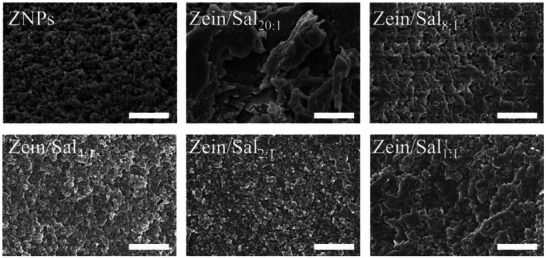
SEM images of ZNPs and various Zein/Sal nanocomposite particles. Scale bars are 1 µm.

Figure 2 displays the morphology of ZNPs and a series of Zein/Sal nanocomposite particles as observed using a scanning electron microscope (SEM). The ZNPs showed a smooth, well‐defined spherical outline, whereas Sal alone displayed a membranous topology (Figure S1). Nanocomposite particles prepared with Zein/Sal mass ratios of 20:1 and 8:1 appeared as big, irregular aggregates. In contrast, Zein/Sal_4:1_ and Zein/Sal_2:1_ showed discrete spherical nanoparticles with distinct boundaries. However, when the Zein/Sal mass ratio reached 1:1, the nanoparticles appeared to adhere to one another, probably due to an excessive amount of Sal. These findings also indicate that Sal concentration influences the preparation of Zein/Sal nanocomposite particles. Based on these results, Zein/Sal_2:1_ was selected for subsequent studies.

### 2.2 Preparation and Formation Mechanism of HPR‐loaded Zein/Sal Nanocomposite Particles

**FIGURE 3:**
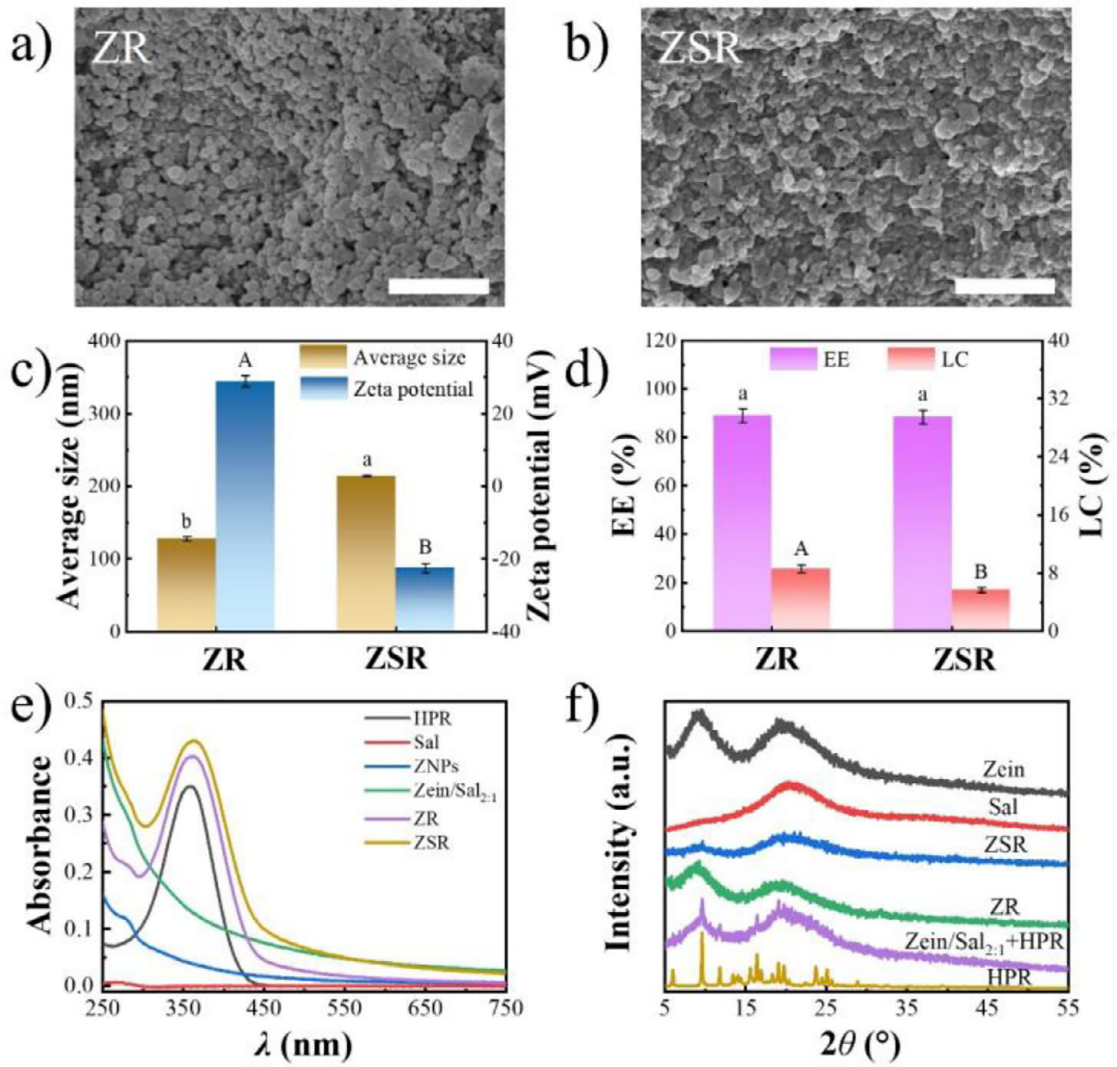
SEM images (a, b), average size and zeta potential (c), EE and LC (d) of ZR and ZSR, respectively; UV–vis spectra of HPR, Sal, ZNPs, Zein/Sal_2:1_, ZR, and ZSR (e); XRD patterns of zein, Sal, ZSR, ZR, HPR, and a physical mixture of Zein/Sal_2:1_ and HPR (f). Different letters represent significant differences (*p* < 0.05). Scale bars are 1 µm.

To examine the encapsulation efficiency (EE) and loading capacity (LC) of Zein/Sal nanocomposite particles, hydroxypinacolone retinoate (HPR) was loaded into Zein/Sal_2:1_, with ZNPs used as the control. The mass ratio between zein and HPR was 10:1, yielding HPR‐loaded Zein/Sal_2:1_ (ZSR) and ZNPs (ZR). As illustrated in Figure 3a–c, both ZR and ZSR kept their spherical morphology when compared with their empty counterparts, but encapsulation changed their average size and zeta potential. The average size of ZNPs increased from 103.36 to 127.76 nm, and the zeta potential decreased slightly from +31.74 to +28.91 mV following HPR loading. Since Sal was coated on the surface of ZR to produce ZSR, the average size of ZSR further increased in comparison to ZR, while the sign of zeta potential was inverted, indicating that the surface of ZSR was successfully coated with Sal. Furthermore, both ZR and ZSR showed high EE values of around 88% (Figure 3d), showing that the presence of Sal did not interfere with the encapsulation of HPR. However, the LC of ZSR was lower than that of ZR, which can be attributed to the higher total mass of the nanocomposite particles.

Spectroscopic and structural analyses further confirmed the successful encapsulation of HPR. As seen in Figure 3e, HPR has a characteristic absorption peak at around 358 nm due to its abundant conjugated double bonds, whereas Sal shows no notable absorption in the range of 250–750 nm [29]. The UV–vis spectra of ZR and ZSR displayed the characteristic absorption of HPR at 358 nm, which was absent in the spectra of carrier counterparts, demonstrating the presence of HPR in the encapsulated form [34]. Figure 3f depicts the x‐ray diffraction (XRD) patterns of zein, Sal, ZSR, ZR, HPR, and a physical mixture of Zein/Sal_2:1_ and HPR. Zein showed two signals at approximately 9.1° and 19.4°, whereas Sal had a single peak at around 20.3°, indicating that both materials were amorphous in nature [35, 36]. HPR exhibited many remarkable diffraction peaks, which corresponded to its crystalline structure. Notably, the typical diffraction peaks of HPR disappeared following encapsulation in ZR and ZSR. However, the diffraction peaks of HPR were detected in the physical mixture of Zein/Sal_2:1_ and HPR. These results indicated that HPR was encapsulated in an amorphous state [37, 38]. This would improve its apparent solubility and bioavailability, as demonstrated before with other hydrophobic compounds encapsulated in zein‐based nanocomposite particles [35, 37].

**FIGURE 4:**
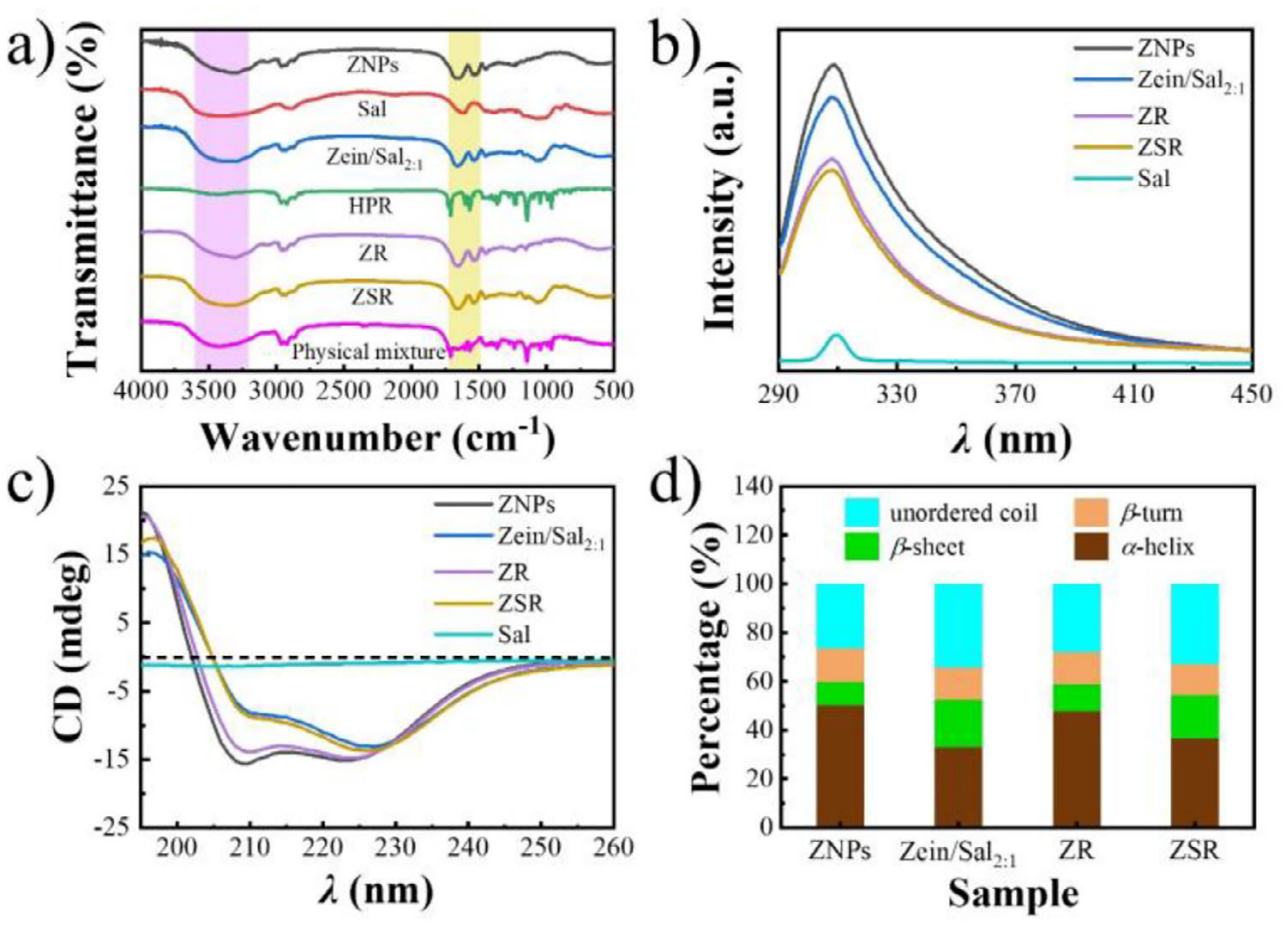
FT‐IR spectra of ZNPs, Sal, Zein/Sal_2:1_, HPR, ZR, ZSR, and a physical mixture of zein, Sal, and HPR (a); fluorescence spectra (b), circular dichroism spectra (c) of ZNPs, Zein/Sal_2:1_, ZR, ZSR, and Sal; secondary structure content (d) of ZNPs, Zein/Sal_2:1_, ZR, and ZSR.

Fourier‐transform infrared (FT‐IR) spectroscopy is often used to analyze the intermolecular interactions between zein and other polymers, as well as zein and lipophilic bioactives. Figure 4a shows that the presence of ─OH causes ZNPs and Sal to have distinct absorption signals in the range of 3100–3500 cm^−1^ [18]. Compared to ZNPs and Sal, the ─OH absorption peak of Zein/Sal_2:1_ shifted from 3306.31 cm^−1^ (ZNPs), 3408.55 cm^−1^ (Sal) to 3336.80 cm^−1^ (Zein/Sal_2:1_), implying that hydrogen bonding interactions exist between zein and Sal [25]. Interestingly, the absorption peaks of amide I (the stretching vibration of C═O) and amide II (the stretching vibration of C─N and the bending vibration of N─H) in Zein/Sal_2:1_ also changed from 1656.14 and 1534.19 cm^−1^ (ZNPs), to 1656.05 and 1540.72 cm^−1^ (Zein/Sal_2:1_), suggesting that hydrophobic interactions exist between zein and Sal, which may be due to the high proportion of hydrophobic amino acids in zein [18]. HPR has no hydroxyl or amino group but two carbonyl groups, so HPR exhibits two characteristic peaks at 1726.91 and 1710.57 cm^−1^, belonging to the asymmetric and symmetric stretching vibration of C═O in HPR [39]. However, these two characteristic peaks of HPR were hardly observed after being encapsulated in ZNPs, and the ─OH, amide I, and amide II absorption peaks of ZNPs changed obviously, shifting from 3306.31, 1656.14, and 1534.19 cm^−1^ (ZNPs), to 3305.70, 1655.71, and 1534.58 cm^−1^ (ZR), implying hydrogen bonding interactions and hydrophobic interactions occurred between zein and HPR [40]. A similar disappearance of HPR's characteristic peaks is also observed in ZSR. In contrast, the characteristic absorption bands of HPR remain clearly visible in the physical mixture of zein, Sal, and HPR, further validating that HPR was effectively encapsulated within ZNPs and Zein/Sal_2:1_ [41].

Figure 4b shows the fluorescence spectra of ZNPs, Zein/Sal_2:1_, ZR, ZSR, and Sal. ZNPs emit a strong fluorescence signal at around 309 nm due to tyrosine residues in zein molecules [42]. Sal did not contain fluorescent groups and only produced a weak fluorescence signal at roughly 309 nm, which might be attributed to the light scattering. The fluorescence intensity of Zein/Sal_2:1_ and ZR was obviously lower than that of ZNPs due to fluorescence quenching effects between zein and Sal/HPR, as these molecular interactions resulted in the fluorescence quenching of ZNPs [43, 44]. The fluorescence intensity of ZSR was the lowest. This was due to the fact that the fluorescence of ZSR was quenched by HPR and Sal simultaneously.

Figure 4c depicts the circular dichroism spectra of ZNPs, Zein/Sal_2:1_, ZR, ZSR, and Sal, while Figure 4d displays the corresponding secondary structure content of zein calculated by CDNN software. The percentages of *α*‐helix, *β*‐sheet, *β*‐turn, and random coil in ZNPs were 50.4%, 9.5%, 13.8%, and 26.3%, respectively, similar to the results reported previously [45]. Sal is a typical polysaccharide without an amide structure; hence, it exhibits no distinct circular dichroic (CD) signal. Compared to ZNPs, the circular dichroic signals of Zein/Sal_2:1_, ZR, and ZSR changed significantly. This could be attributed to the intermolecular interactions between zein and Sal, as well as between zein and HPR, both of which affect the secondary structure of zein [45]. These results were consistent with the results of FT‐IR and fluorescence spectroscopy, and further confirmed the interactions between zein and HPR, as well as zein and Sal.

### 2.3 Colloidal Stability of ZSR

**FIGURE 5:**
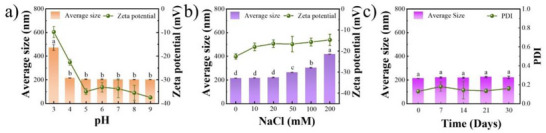
Changes in average size, zeta potential, or PDI of ZSR at varying pH (a), ionic strength (b), and room temperature storage time (c). Different letters represent significant differences (*p* < 0.05).

The practical use of nanoparticles as delivery vehicles requires adequate colloidal stability under a series of environmental conditions. As a result, the dispersion of ZSR was assessed in terms of changes in pH, ionic strength, and storage time, using ZR as the control group. As shown in Figure S2, ZR had poor pH stability, with visible precipitation occurring at pH levels ranging from 5 to 7, close to the isoelectric point of zein. In contrast, ZSR remained stable over a wide pH range (4.0 to 9.0), with no significant changes in particle size (Figure 5a) or apparent aggregation (Figure S3). At pH 4.0, ZSR showed a certain reduction in zeta potential, which could be due to the protonation of carboxyl groups on Sal, yet the average particle size of ZSR remained constant [16]. This was due to Sal, which provided steric stabilization while counteracting the lower electrostatic repulsion. Only at pH 3.0 did ZSR exhibit an increase in average particle size. These results revealed that the incorporation of Sal greatly increased the pH tolerance of the nanoparticles, consistent with previous findings on other polysaccharides [19]. In regard to ionic stability, ZR precipitated quickly at NaCl concentrations as low as 20 mM (Figure S4), but ZSR was colloidally stable below 100 mM (Figures 5b and S5). The improved salt resistance of ZSR was attributed to the hydrophilic Sal coating, which provides steric hindrance while encountering a screening effect of salt ions on surface charges. The long‐term storage stability of ZSR was also assessed at room temperature (Figure 5c). Notably, ZSR remained well‐dispersed and consistent in size after 30 days. This further validates the stabilizing effect of Sal. In a word, these results demonstrated that coating ZNPs with Sal significantly increased their colloidal stability. This improvement would make Zein/Sal nanocomposite particles useful as a delivery system for lipophilic bioactives.

### 2.4 Protection of HPR From Environmental Stress

**FIGURE 6:**
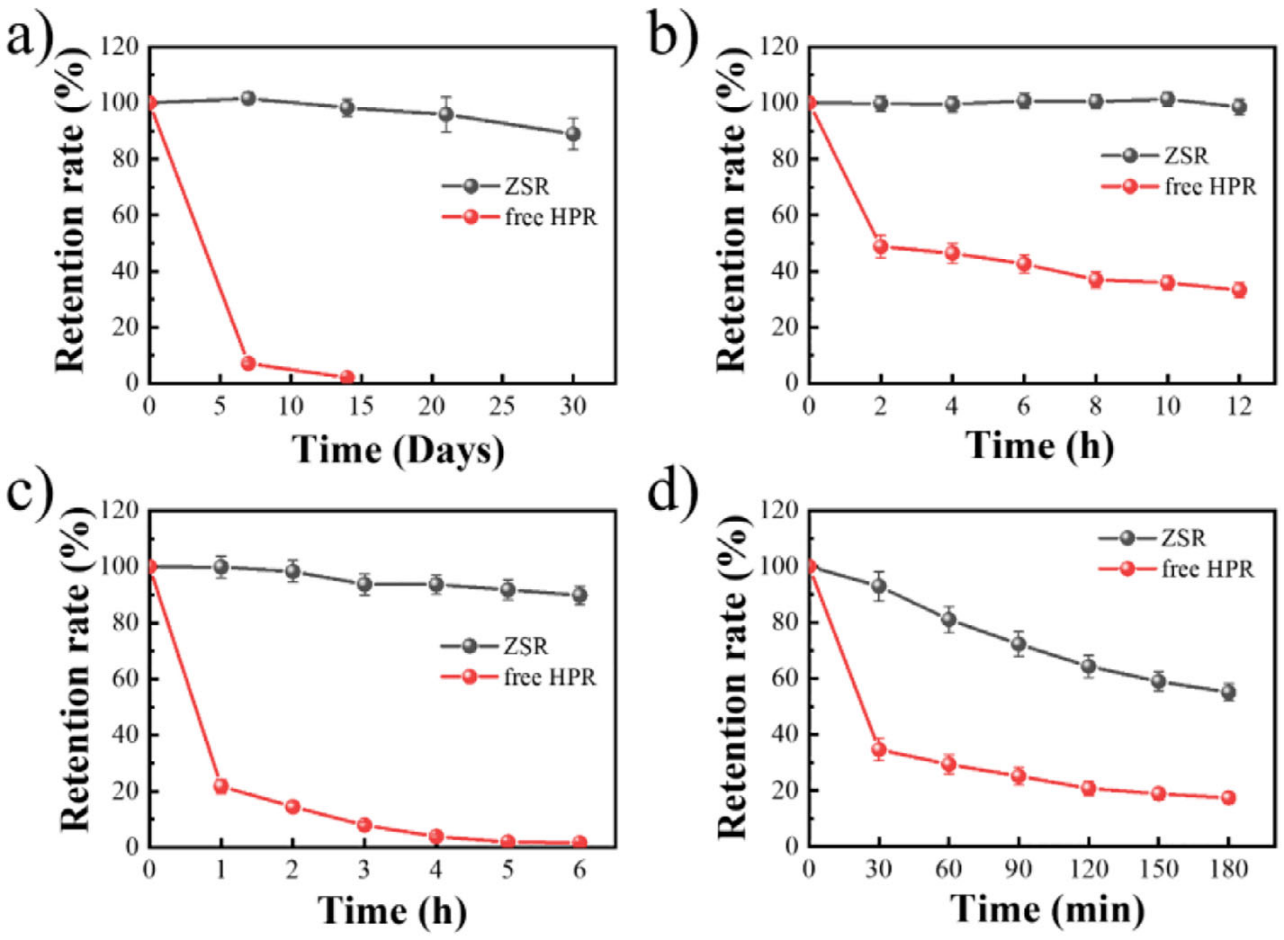
Retention rate of HPR over time at room temperature (a), 37°C (b), 80°C (c), and light irradiation (d).

HPR is chemically unstable because of its conjugated double bonds, making it susceptible to degradation under various environmental conditions [29]. Figure 6 shows the stability of HPR encapsulated in Zein/Sal_2:1_ and free HPR. Figure 6a demonstrates that free HPR degraded rapidly at room temperature, with the retention rate dropping to only 7.12% after 7 days. In contrast, HPR encapsulated in Zein/Sal_2:1_ remained impressively stable, with a retention rate of more than 85% even after 30 days, demonstrating the efficacy of zein‐based carriers in protecting against oxidative and moisture‐induced degradation [46]. When the temperature was elevated to physiological and accelerated settings (37°C and 80°C), as shown in Figure 6b,c, free HPR degraded dramatically, leaving only 48.84% and 14.45% after 2 h at 37°C and 80°C, respectively. In contrast, Zein/Sal_2:1_ effectively reduced heat‐induced degradation: more than 98% of HPR remained after 12 h at 37°C and more than 85% after 6 h at 80°C. Furthermore, after light treatment (Figure 6d), free HPR degraded rapidly to 34.60% within 30 min, whereas Zein/Sal_2:1_ provided good photoprotection, with more than 50% of HPR remaining after 180 min. Overall, these results demonstrate that encapsulating HPR in carrier particles significantly enhances its stability, indicating a promising approach for preserving its efficacy in potential applications.

### 2.5 Cytotoxicity, Cellular Uptake, and Cell Migration

**FIGURE 7:**
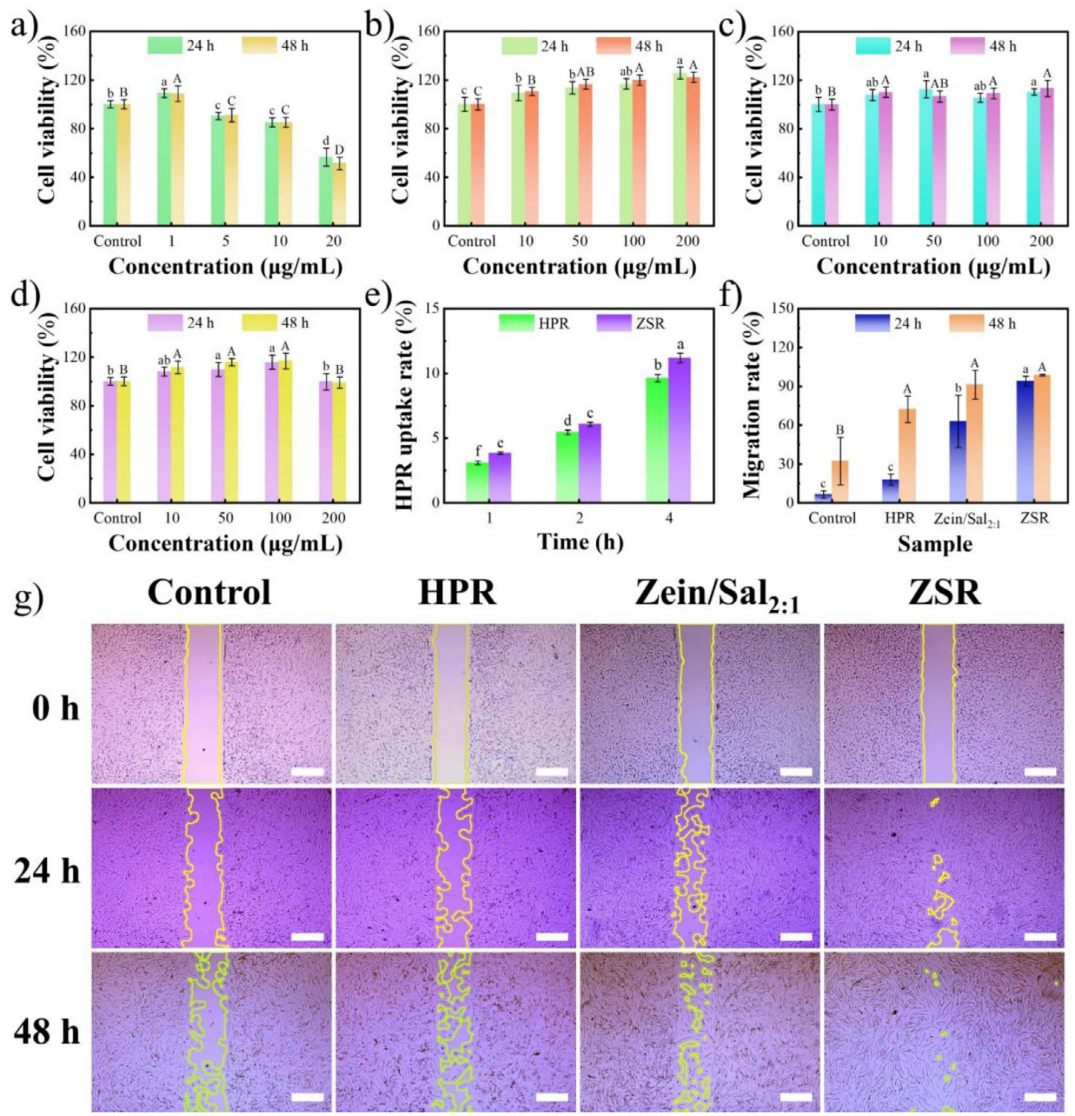
Cell viability of HFF‐1 cells treated with different concentrations of HPR (a), Zein/Sal_2:1_ (b), Sal (c), and ZSR (d); cellular uptake rate of HPR and ZSR (e); cell migration rate (f), and representative digital images of cell migration (g) of HFF‐1 cells treated without (control group) or with HPR, Zein/Sal_2:1_, and ZSR. Different letters represent significant differences (*p* < 0.05). Scale bars are 400 µm.

The cytotoxicity, cellular uptake, and cell migration assays were performed using human foreskin fibroblasts (HFF‐1). As shown in Figure 7a, low‐dose HPR (1 µg/mL) promoted cell proliferation, with cell viabilities of 109.15% and 108.79% at 24 h and 48 h, respectively. In contrast, high‐dose HPR (20 µg/mL) caused severe cytotoxicity, resulting in cell viabilities of 56.53% at 24 h and 51.32% at 48 h. Zein/Sal_2:1_ was highly biocompatible and promoted cell proliferation by up to 125.71% at 24 h and 122.20% at 48 h at 200 µg/mL (Figure 7b). A similar trend was observed with Sal alone (Figure 7c), consistent with previous reports that *β*‐glucans enhance cell proliferation in wound healing [47, 48]. Remarkably, ZSR increased cell viability when compared to HPR alone (Figure 7d). This shows that encapsulation can reduce the cytotoxicity of HPR. Notably, zein/chondroitin sulfate nanocomposite particles were cytocompatible with human renal epithelial cells but did not promote cell proliferation [49]. Similar results were seen with zein/fucoidan nanocomposite particles [42]. These results further demonstrate that Sal not only enhances the colloidal stability of zein‐based nanoparticles but also imparts a distinct effect on cell proliferation.

As shown in Figure 7e, Zein/Sal_2:1_ enhanced the cellular uptake of HPR. Specifically, the intracellular HPR content in HFF‐1 cells treated with ZSR was significantly higher (*p* < 0.05) than that of free HPR at all investigated incubation times (1, 2, and 4 h). The enhanced cellular uptake of HPR by Zein/Sal_2:1_ could be attributed to improving the intrinsic limitations of HPR in solubility and stability, optimizing carrier‐cell surface interactions, and exploiting the size‐dependent advantages of nanoparticle‐mediated endocytosis [50, 51]. As for cell migration (Figure 7f,g), HPR (10 µg/mL) had little migration‐promoting ability at 24 h, while Zein/Sal_2:1_ promoted cell migration significantly at 24 h (*p* < 0.05). ZSR exhibited the highest migration rate (93.86% after 24 h), implying that the combination of HPR and Zein/Sal_2:1_ had a synergistic effect on cell motility. Notably, prolonged incubation generally promoted cell migration for all samples. However, the overall trend remained consistent with that observed at 24 h, with the ZSR group still exhibiting the highest cell migration rate after 48 h. These findings suggested that Zein/Sal_2:1_ not only improves the safety of HPR but also enhances its regeneration efficacy, making ZSR an attractive candidate for cutaneous applications.

### 2.6 In Vitro Release and In Vitro Transdermal Delivery of HPR

**FIGURE 8:**
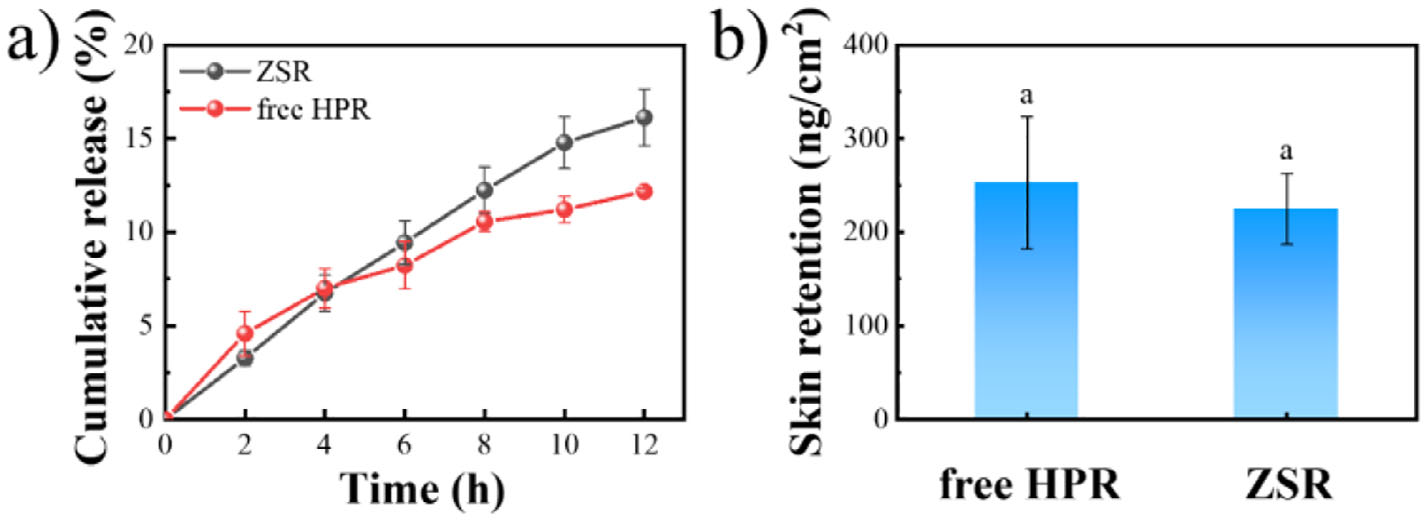
In vitro release profiles (a) and skin retention (b) of HPR in free and in ZSR. The same letters indicate no significant differences (*p* > 0.05).

The in vitro release and skin penetration of encapsulated and free HPR were evaluated using the Franz diffusion cell. Figure 8a depicts the results of the in vitro release assay. ZSR showed a lower cumulative release degree (3.28%) within the first 2 h compared to free HPR (4.58%). This can be attributed to the encapsulation, which delayed the release of HPR in ZSR. After 4 h, however, the cumulative release degree of HPR from ZSR exceeded that of free HPR, most likely because free HPR degraded rapidly at 37°C (Figure 6b), whereas encapsulated HPR remained stable and diffused continuously through the Sal coating. In the transdermal experiment, HPR was detected only in the skin layer but not in the release medium after 12 h, indicating a limited penetration depth of HPR. Figure 8b exhibits that the ZSR group had a smaller amount of HPR in the skin compared to the free HPR group, but there was no significant difference (*p *> 0.05). The result was contrary to the in vitro release assay, which may be due to the use of isosorbide dimethyl ether (DMI) in the free HPR group, a skin penetration enhancer for HPR, which promoted the skin penetration of HPR [52].

## 3 Conclusions

In summary, Zein/Sal nanocomposite particles were developed using antisolvent precipitation followed by a polysaccharide coating. An optimal Zein/Sal mass ratio of 2:1 produced well‐dispersed, homogeneous Zein/Sal nanocomposite particles with an average size of about 238.66 nm and a zeta potential of −24.34 mV. The electrostatic interactions, hydrogen bonding, and hydrophobic interactions between ZNPs and Sal were responsible for the formation of Zein/Sal nanocomposite particles. In addition, amorphous HPR was encapsulated into Zein/Sal nanocomposite particles via hydrogen bonding and hydrophobic interactions between zein and HPR, with a relatively high EE of about 88%. Incorporation of Sal remarkably enhanced the colloidal stability: HPR‐loaded Zein/Sal nanocomposite particles remained stable across pH ranges of 4.0 to 9.0, tolerated ionic strengths of up to 100 mM NaCl, and maintained an unchanged average particle size after 30 days of storage at room temperature. Encapsulation also gave HPR good thermal and light stress resistance, demonstrating the outstanding stabilizing capability of carrier particles. Furthermore, the Zein/Sal nanocomposite particles were highly biocompatible, enhancing the viability of HFF‐1 cells up to 122.20% after 48 h at 200 µg/mL. Encapsulation reduced the cytotoxicity of HPR and increased cellular uptake and migration of HFF‐1 cells, indicating synergistic regenerative effects resulting from HPR and Zein/Sal_2:1_. Furthermore, in vitro release and delivery assays showed a sustained release and effective transdermal penetration of encapsulated HPR. Overall, these results highlight Sal as a functional stabilizer that enhances the colloidal stability and biological performance of zein‐based carriers, offering a promising and versatile platform for the delivery of lipophilic bioactives in pharmaceutical, food, and cosmetic applications.

## 4 Experimental Section

### 4.1 Materials

Salecan (Sal, 2000 kDa) was purchased from Sichuan Synlight Biotech Co., Ltd. (China). Zein (Z3625) was obtained from Sigma–Aldrich (USA). Hydroxypinacolone retinoate (HPR) was supplied by Yuanye Biotech Co., Ltd. (China). NaOH, HCl, NaCl, potassium bromide, potassium chloride, dipotassium hydrogen phosphate, and ethanol were provided by Sinopharm (China). Ethanol (HPLC grade), acetic acid (HPLC grade), Tween‐20, and Tween‐80 were purchased from Titan Scientific Co., Ltd. (China). 8‐Anilino‐1‐naphthalenesulfonic acid (ANS) was obtained from Meryer Technologies Co., Ltd. (China). Isosorbide dimethyl ether (DMI) was purchased from Macklin (China). Human foreskin fibroblasts (HFF‐1) were obtained from BeNa Culture Collection (China). The Cell Counting Kit‐8 (CCK‐8) was supplied by Beyotime Biotechnology (China). Dulbecco's Modified Eagle Medium (DMEM) was supplied by Cytiva (China). Phosphate Buffered Saline (PBS) and 0.25% Trypsin‐EDTA were purchased from Gibco (China).

### 4.2 Preparation of Zein/Sal Nanocomposite Particles Loaded With or Without HPR

Zein nanoparticles (ZNPs) were first prepared using the antisolvent precipitation method [53]. Briefly, 100 mg of zein powder was dissolved in 10 mL of aqueous ethanol (85 vol%) before being immediately poured into 30 mL of deionized water (pH 4.0). The resulting dispersion was subjected to rotary evaporation (35°C, −0.1 MPa) to remove ethanol and part of the water, yielding the aqueous dispersion of ZNPs. To prepare Zein/Sal nanocomposite particles, 20 mL of the prepared aqueous dispersion of ZNPs was mixed with 80 mL of Sal solution at different concentrations. The resulting samples were denoted as Zein/Sal_20:1_, Zein/Sal_8:1_, Zein/Sal_4:1_, Zein/Sal_2:1_, and Zein/Sal_1:1_, based on the mass ratio of zein to Sal. Portions of each dispersion were lyophilized to produce powders for subsequent characterization. HPR‐loaded Zein/Sal nanocomposite particles (ZSR) and ZNPs (ZR) were prepared using the same process as before, with the exception that 10 mg of HPR was added to the zein solution before particle production.

The encapsulation efficiency (EE) and loading capacity (LC) of HPR were determined via high‐performance liquid chromatography (HPLC), using a Waters Arc HPLC system (USA) equipped with a Symmetry C18 column (4.6 × 250 mm, 5 µm). The mobile phase consisted of ethanol and aqueous acetic acid (0.5 wt%) at a volume ratio of 85:15. The flow rate and column temperature were set at 0.8 mL/min and 30°C, respectively. The detection wavelength, injection volume, and total run time were 358 nm, 10 µL, and 12.5 min, respectively. HPR concentration was determined using the standard curve equation: *y* = 60887100*x*, *R* ^2^ = 0.9999, where *x* is the concentration of HPR (mg/mL), and *y* is the corresponding peak area (µV·s). The standard curve of HPR is shown in Figure S6. EE and LC were calculated as follows:

(1) $$\mathrm{EE}\;(\%)=\frac{\mathrm{mass}\;\mathrm{of}\;\mathrm{encapsulated}\;\mathrm{HPR}}{\mathrm{mass}\;\mathrm{of}\;\mathrm{HPR}\;\mathrm{input}}\;\times \;100\%$$

(2) $$\mathrm{LC}\;(\%)=\frac{\mathrm{mass}\;\mathrm{of}\;\mathrm{encapsulated}\;\mathrm{HPR}}{\mathrm{mass}\;\mathrm{of}\;\mathrm{zein}\;\mathrm{and}\;\mathrm{Sal}\;\mathrm{input}}\;\times \;100\%$$

### 4.3 Characterization of ZSR and ZR

The particle size, polydispersity index (PDI), and zeta potential of samples were analyzed using a NanoBrook Omni analyzer (Brookhaven, USA). The morphology of samples was observed using a Hitachi S‐4800 (Japan) scanning electron microscope (SEM) following gold sputter‐coating. The surface hydrophobicity index (H_0_) was evaluated using ANS as a fluorescent probe [32]. The experiment involved adding 40 µL of 2 mM ANS solution (10 mM PBS, pH 7.0) to 8 mL of particle dispersion at various concentrations and incubating for 20 min in the dark. The fluorescence emission spectra were collected using an F‐7000 fluorescence spectrophotometer (Hitachi, Japan) with an excitation wavelength of 370 nm and an emission wavelength range of 400–600 nm. Both the excitation and emission slit widths were set at 5 nm, and the scanning speed was 1200 nm/min. The slope of the linear regression plot of fluorescence intensity at 470 nm against zein concentration was designated as H_0_. The secondary structure of zein was analyzed using a Chirascan V100 circular dichroism spectrometer (Applied Photophysics, UK) with the following parameters: particle concentration 1 mg/mL (based on zein), path length 0.1 mm, scanning range 195–260 nm, bandwidth 1 nm, scanning speed 1 nm/s, and 1 s per point. The secondary structural composition was calculated using the CDNN software. The fluorescence emission spectra of as‐prepared particles were collected at excitation and emission wavelengths of 280 nm and 290–450 nm, respectively. The UV‐Vis absorption spectra of aqueous particle dispersions were recorded using a TU‐1950 spectrophotometer (Persee, China). X‐ray diffraction (XRD) patterns were obtained using an AXS D8 diffractometer (Bruker, Germany) with a scanning range of 5–55°and a scanning speed of 5°/min. Fourier‐transform infrared (FT‐IR) spectra were collected using a Nicolet iS5 spectrometer (Thermo Fisher Scientific, USA) over a wavenumber range of 4000–500 cm^−1^ with 64 scans and a resolution of 4 cm^−1^.

### 4.4 Colloidal Stability of ZSR and ZR

The colloidal stability of ZSR and ZR was tested at various pH values and ionic concentrations [16]. The aqueous dispersions of ZSR and ZR were adjusted to pH values ranging from 3.0 to 9.0 and held at room temperature for 24 h. To assess ionic stability, varying volumes of 4 M NaCl solution were added to the aqueous dispersions of ZSR and ZR to yield different ionic strengths, followed by incubation at room temperature for 24 h. After that, the average particle size and zeta potential of ZSR and ZR were determined. To evaluate long‐term storage stability, the aqueous dispersions of ZSR were maintained at room temperature, and samples were collected at predetermined time intervals to measure changes in particle size and PDI.

### 4.5 Stability of Encapsulated and Free HPR

The stability of HPR encapsulated in Zein/Sal_2:1_ and free HPR (dissolved in a 10 wt% DMI aqueous solution) was investigated at various temperature conditions and light exposure. To conduct thermal stability assays, samples were kept at room temperature, 37°C and 80°C, respectively. To assess photochemical stability, samples were exposed to a light cabinet (14 W). The concentration of HPR was quantified at regular intervals using the above‐mentioned HPLC method. The retention rate of HPR was calculated using the following equation:

(3) $$\mathrm{Retention}\;\mathrm{rate}\;\mathrm{of}\;\mathrm{HPR}\;(\%)=\frac{C_{t}}{C_{0}}\;\times \;100\%$$

where *C*_t_ is the concentration of HPR after treatment, and *C*_0_ is the concentration of HPR initial.

### 4.6 Cytotoxicity, Cellular Uptake, and Cell Migration Assays

The cytotoxicity of HPR, Sal, Zein/Sal_2:1_, and ZSR was assessed via the CCK‐8 assay using HFF‐1 cells [42]. HFF‐1 cells were seeded in 96‐well plates at a density of 1 × 10^4^ cells per well and incubated for 24 h (37°C and 5% CO_2_). After removing the culture medium and washing with PBS, 100 µL of sample solutions at different concentrations were added to each well and cultured for 24 or 48 h. After removing the samples, the cells were washed twice with PBS before adding CCK‐8 reagent (10 µL) and fresh culture medium (90 µL) to each well. After 90 min of incubation at 37°C, the absorbance at 450 nm was measured, and cell viability was calculated as follows:

(4) $$\mathrm{Cell}\;\mathrm{viability}\;(\%)=\frac{A_{S}-A_{B}}{A_{C}-A_{B}}\;\times \;100\%$$

where *A*_B_, *A*_C_, and *A*_S_ are the absorbance of the blank, control, and sample groups, respectively.

The cellular uptake assay was performed following a previously reported method [50, 54]. HFF‐1 cells were seeded in 12‐well plates at a density of 1 × 10^5^ cells per well and cultured at 37°C with 5% CO_2_ for 48 h to promote cell adhesion and reach over 80% confluence. Subsequently, the cells were treated with 1 mL of culture medium with HPR (10 µg/mL). After incubation for 1, 2, and 4 h, respectively, the supernatant was removed, and the cells were rinsed twice with PBS to remove unabsorbed HPR. Next, 0.2 mL of lysis buffer (0.025% trypsin and 1% Tween‐20 in PBS) was added to each well, followed by 30 min of incubation at 37°C to lyse cells. Then, 2.8 mL of ethanol was added, and the mixture was sonicated for 10 min to fully extract intracellular HPR. Finally, the extracted HPR content was measured by HPLC.

(5) $$\mathrm{HPR}\;\mathrm{uptake}\;\mathrm{rate}\;(\%)\;=\frac{m_{t}}{m_{0}}\;\times 100\%$$

where, *m*_t_ and *m*_0_ represent the mass of HPR in HFF‐1 cells and the initial mass of HPR, respectively.

The effect of ZSR, Zein/Sal_2:1_, and HPR on cell migration was evaluated using the scratch assay. In a 6‐well plate, cell culture inserts were placed in each well, and HFF‐1 cells were seeded at a density of 2 × 10^4^ cells per insert. After 48 h of incubation (37°C, 5% CO_2_), the culture medium was withdrawn, and the cells were washed with PBS. Subsequently, each sample dispersion (2 mL) was added to the wells, followed by incubation for 24 or 48 h (37°C, 5% CO_2_). Cell migration was studied using an optical microscope, and images were captured to evaluate cell migration behavior. The scratch area was calculated via ImageJ software, while the cell migration rate was calculated according to the following equation:

(6) $$\mathrm{Migration}\;\mathrm{rate}\;(\%)=\frac{S_{0}-S_{t}}{S_{0}}\;\times \;100\%$$

where *S*_0_ and *S*_t_ are the scratch areas of initial and after 24 or 48 h, respectively.

### 4.7 In Vitro Release and Transdermal Delivery

In vitro release of HPR from ZSR was investigated using a Franz diffusion cell, with free HPR (dissolved in a 10 wt% DMI aqueous solution) as the control. 2 mL of each sample was added to the donor chamber, while the receptor chamber was filled with 8 mL of release medium (5% Tween‐80, 10 mM PBS, 137 mM NaCl, and 2.7 mM KCl, pH 7.4). The two chambers were separated by a polyvinylidene fluoride (PVDF) membrane with a pore size of 0.1 µm. The system was maintained at 37°C, and the release medium was stirred (400 rpm) for 12 h. Every 2 h, the release medium (200 µL) was withdrawn and replaced with an equal amount of fresh medium. The HPR concentration was quantified by HPLC as described previously, and the cumulative release rate of HPR was calculated using:

(7) $$\mathrm{Cumulative}\;\mathrm{release}\;\mathrm{rate}\;\mathrm{of}\;\mathrm{HPR}\;(\%)=\frac{V_{1}C_{n}+\sum_{i\;=1}^{n-1}V_{2}C_{i}}{m_{0}}\;\times \;100\%$$

where *V*_1_ and *V*_2_ represent the volume of the receptor chamber (8 mL) and collected samples (0.2 mL), respectively; *C*_n_ and *C*_i_ are the concentration of HPR at each sampling time and each *n*‐1 sampling time, respectively; *m*_0_ is the initial mass of HPR loaded into the donor chamber.

Under identical settings, the in vitro transdermal study used porcine skin instead of PVDF membrane. Following the diffusion test, the skin was rinsed with PBS, sliced into small pieces, and ultrasonicated with ethanol (3 mL) for 30 min to extract HPR. The HPR concentration in the extracts was measured using the previously mentioned HPLC method. The skin retention of HPR was calculated as follows:

(8) $$\mathrm{Skin}\;\mathrm{retention}\;(\mathrm{ng}/cm^{2})=\frac{\mathrm{mass}\;\mathrm{of}\;\mathrm{HPR}\;\mathrm{in}\;\mathrm{skin}}{\mathrm{transdermal}\;\mathrm{area}}$$

### 4.8 Statistical Analysis

Experiments were done in triplicate, while results were reported as mean ± SD. One‐way analysis of variance (ANOVA) and Tukey's test were used for significance analysis. *p* < 0.05 indicated statistical significance.

## Conflicts of Interest

The authors declare no conflicts of interest.

## Supporting information




**Supporting File**: asia70741‐sup‐0001‐SuppMat.docx.

## Data Availability

The data that support the findings of this study are available on request from the corresponding author. The data are not publicly available due to privacy or ethical restrictions.
