# Supplementary material for: Preparation and Characterization of Zein/Salecan Nanocomposite Particles for Enhanced Stability and Bioactivity of Lipophilic Retinoids
Source: Chem Asian J. 2026 Apr 14;21:e70741. doi: 10.1002/asia.70741 (PMC13077527; doi:10.1002/asia.70741)
Supplement: Supplementary file 1 — Supporting File: asia70741‐sup‐0001‐SuppMat.docx. [file ASIA-21-e70741-s001.docx]

**Supporting Information:**

**Preparation and characterization of zein/salecan nanocomposite particles for enhanced stability and bioactivity of lipophilic retinoids**

Jie Mei,^[a]^ Chen Li,^[b]^ Yunxing Li,* ^[a]^ Beizhe Chang,^[a]^ Hang Jiang,^[a]^ Cheng Yang,*^[a]^ Bingtian Zhao,^[a]^ To Ngai,*^[c]^

[a] *Dr. J. Mei, Prof. Y. Li, Dr. B. Chang, Prof. H. Jiang, Prof. C. Yang*, *Prof. B. Zhao*
*Key Laboratory of Synthetic and Biological Colloids, Ministry of Education, School of Chemical and Material Engineering, Jiangnan University, Wuxi 214122, China.
E-mail:* *[yunxingli@jiangnan.edu.cn](mailto:yunxingli@jiangnan.edu.cn);* [*cyang@jiangnan.edu.cn*](mailto:cyang@jiangnan.edu.cn)

[b] *Dr. C. Li*
*School of Chemistry and Environmental Engineering, Yuxi Normal University, Yuxi 653100, China.*

[c] *Prof. T.* Ngai
*Department of Chemistry, The Chinese University of Hong Kong, Shatin, N. T., Hong Kong, China.
E-mail:* [*tongai@cuhk.edu.hk*](mailto:tongai@cuhk.edu.hk)


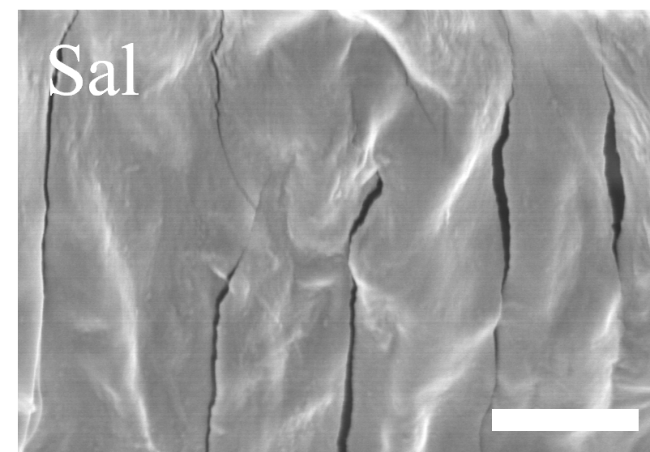


**Figure S1.** SEM image of Sal. Scale bar is 1 μm.


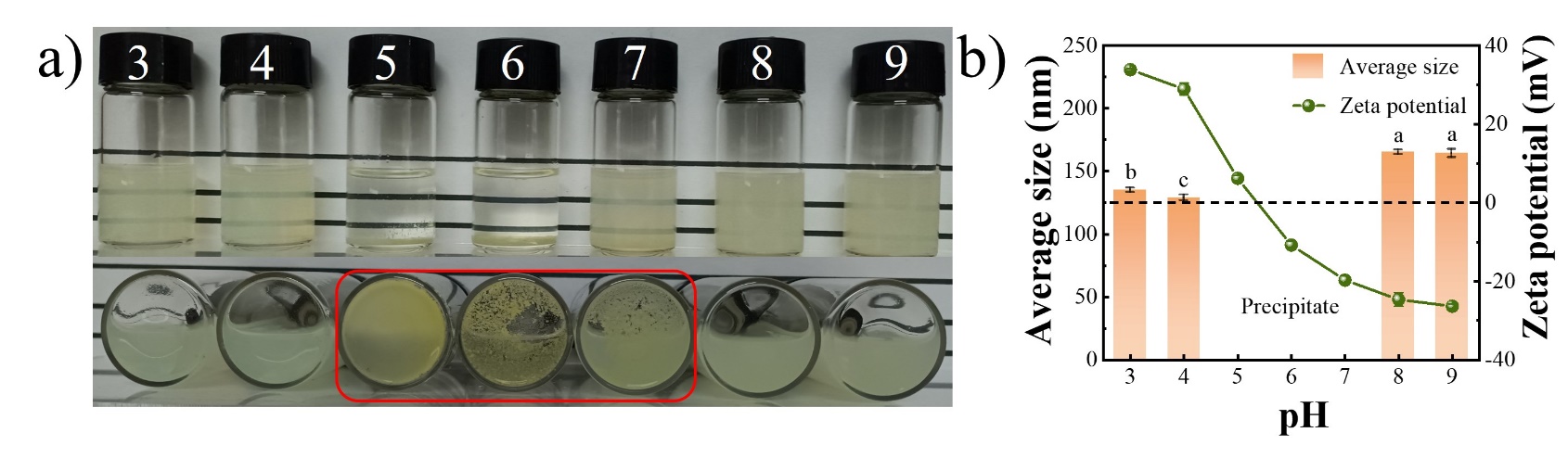


**Figure S2.** Digital image of aqueous dispersions of ZR at different pH (a), average size and zeta potential of ZR at different pH (b). Different letters represent significant differences (*p* < 0.05).


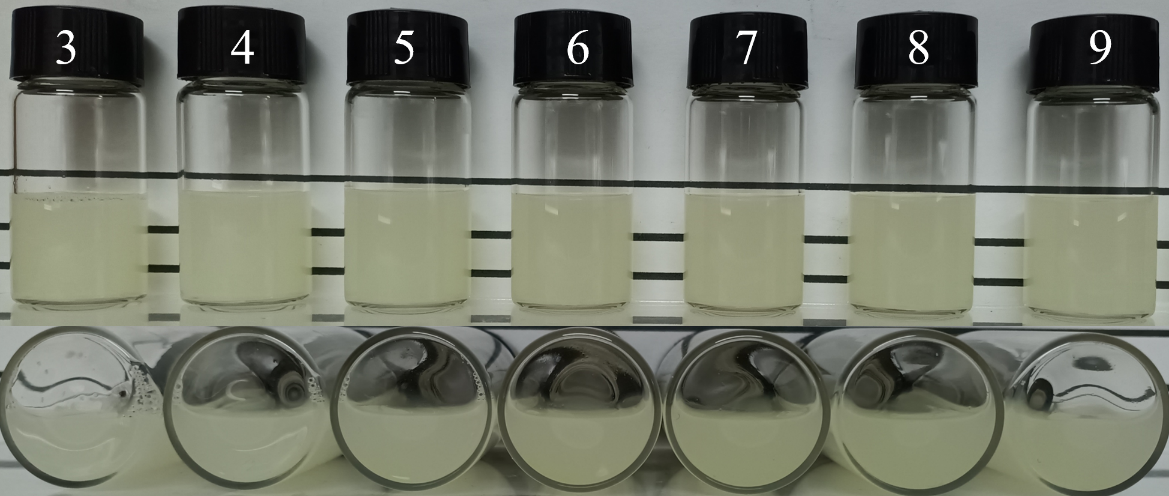


**Figure S3.** Digital image of aqueous dispersions of ZSR at different pH.


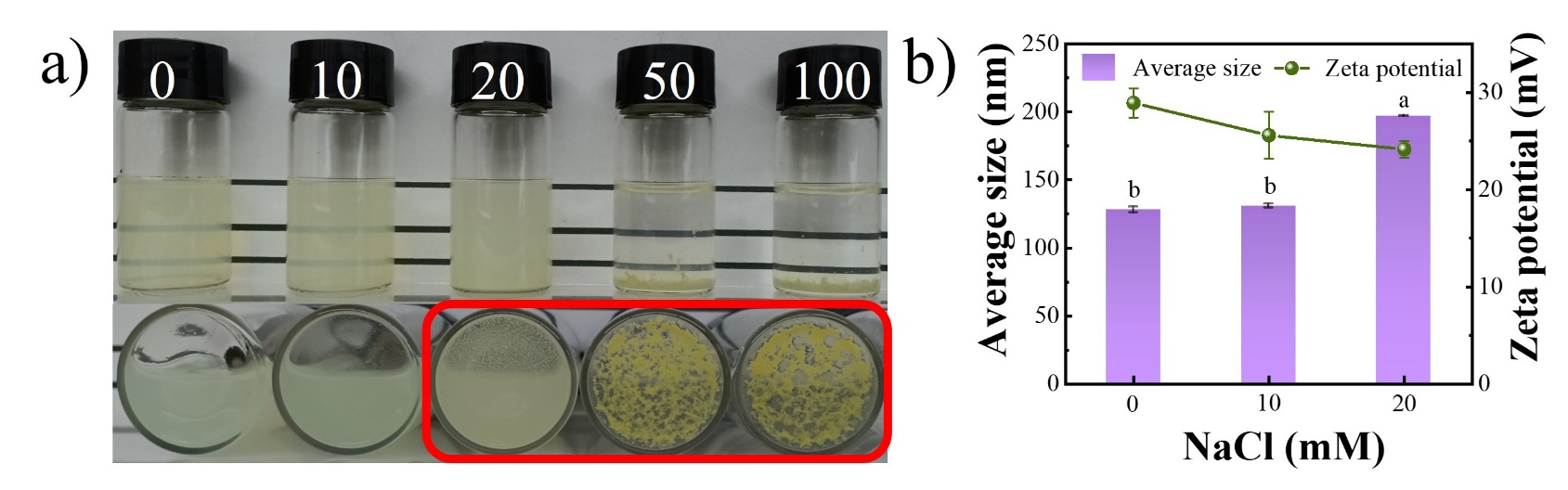


**Figure S4.** Digital image of aqueous dispersions of ZR at different concentration of NaCl (mM) (a), average size and zeta potential of ZR at different concentration of NaCl (mM) (b). Different letters represent significant differences (*p* < 0.05).


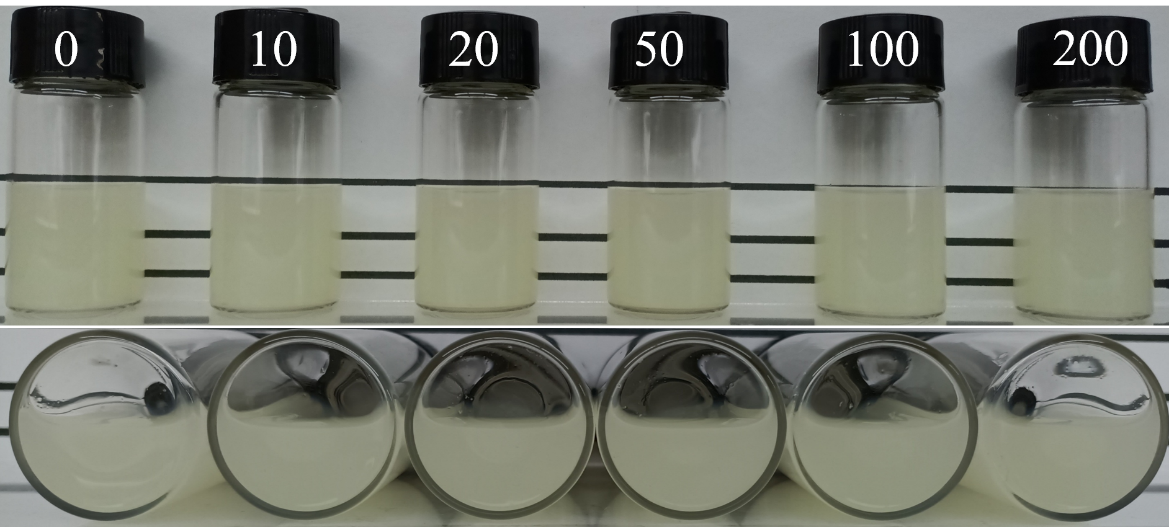


**Figure S5.** Digital image of aqueous dispersions of ZSR at different concentration of NaCl (mM).


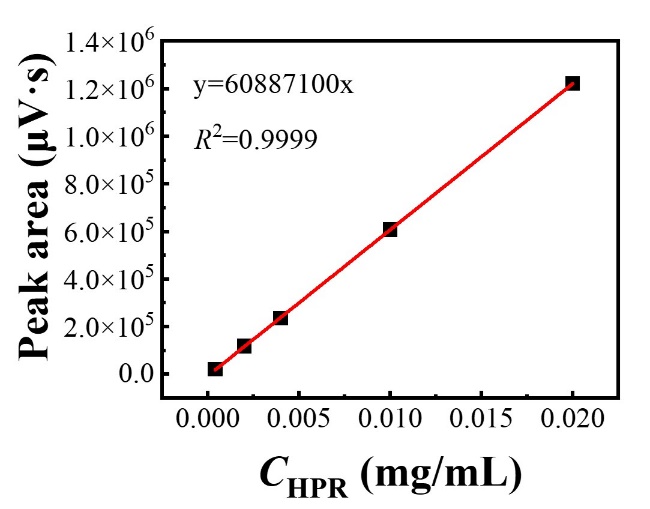


**Figure S6.** Standard curve for the determination of HPR concentration.
